# Early mobilization does not reduce the risk of deep venous thrombosis after Achilles tendon rupture: a randomized controlled trial

**DOI:** 10.1007/s00167-019-05767-x

**Published:** 2019-11-02

**Authors:** Susanna Aufwerber, Annette Heijne, Gunnar Edman, Karin Grävare Silbernagel, Paul W. Ackermann

**Affiliations:** 1 Department of Molecular Medicine and Surgery, Karolinska Institutet, Stockholm, Sweden; 2 Functional Area Occupational Therapy and Physiotherapy, Allied Health Professionals Function, A6:U1, Karolinska University Hospital, 171 76 Stockholm, Sweden; 3 Department of Neurobiology, Care Sciences and Society, Division of Physiotherapy, Karolinska Institutet, Huddinge, Sweden; 4 R&D, Norrtälje Hospital, Tiohundra AB, Norrtälje, Sweden; 5 Department of Clinical Sciences, Karolinska Institutet, Stockholm, Sweden; 6 Department of Physical Therapy, University of Delaware, Newark, DE USA; 7 Department of Orthopedic Surgery, Karolinska University Hospital, Stockholm, Sweden

**Keywords:** Achilles tendon, Rupture, Venous thrombosis, Weightbearing, Duplex ultrasonography

## Abstract

**Purpose:**

The hypothesis was that early functional mobilization would reduce the incidence of deep venous thrombosis (DVT) during leg immobilization after Achilles tendon rupture surgery. A secondary aim was to evaluate if the amount of weightbearing and daily steps influenced the risk of sustaining a DVT.

**Methods:**

One-hundred and fifty patients with Achilles tendon rupture repair were randomized to treatment with early functional mobilization, encouraging full weightbearing and ankle motion in orthosis, or treatment-as-usual, i.e., 2 weeks of unloading in plaster cast followed by 4 weeks weightbearing in orthosis. At 2 and 6 weeks postoperatively, all patients were screened for DVT using compression duplex ultrasound. During the first 2 weeks postoperatively, patient-reported loading, pain and step counts were assessed.

**Results:**

At 2 weeks, 28/96 (29%) of the patients in early functional mobilization group and 15/49 (31%) in the control group (n.s) had sustained a DVT. At 6 weeks, the DVT rate was 35/94 (37%) in the early functional mobilization and 14/49 (29%) in the control group (n.s). During the first postoperative week, the early functional mobilization group reported low loading and higher experience of pain vs. the control group (*p* = 0.001). Low patient-reported loading ≤ 50% (OR = 4.3; 95% CI 1.28–14.3) was found to be an independent risk factor for DVT, in addition to high BMI and higher age.

**Conclusions:**

Early functional mobilization does not prevent the high incidence of DVT during leg immobilization in patients with Achilles tendon rupture as compared to treatment-as-usual. The low efficacy of early functional mobilization is mainly explained by postoperative pain and subsequent low weightbearing. To minimize the risk of DVT, patients should be encouraged to load at least 50% of body weight on the injured leg 1 week after surgery.

**Level of evidence:**

Therapeutic, level 1

## Introduction

Achilles tendon rupture (ATR) is the sports-related injury associated with the highest risk of deep venous thrombosis (DVT), approximately 35–50%, irrespective of operative or non-operative treatment [4, 7, 8, 15, 19]. Immobilization of the lower limb in a plaster cast or brace is considered the triggering factor for venous thromboembolism (VTE). A highly effective DVT preventive intervention during leg immobilization after ATR is not yet developed [22].

Chemoprophylaxis with low molecular weight heparin (LMWH) has, in leg immobilized patients, shown only to give a small reduction of VTE [22], and further, to be non-effective in ATR patients [4, 15]. The reason for the poor preventive effect of LMHW and, therefore, the inability to stop the formation of DVT is presumably an insufficient blood circulation during immobilization of the limb. Hence, other means to increase venous return during leg immobilization are warranted.

Active ankle and toe range of motion exercises during limb immobilization increase the venous blood flow in the lower limb [9, 13, 21]. Venous return is moreover significantly higher with partial or full weightbearing compared to non-weightbearing, and with the ankle in a neutral position compared to plantar flexion [5]. Theoretically, therefore, weightbearing and ankle motion during orthosis immobilization could reduce the risk of DVT.

Early functional mobilization (EFM), i.e., immediate postoperative weightbearing and ankle motion, has shown greater patient satisfaction compared to immobilization in ATR patients [10, 16, 18]. However, whether EFM can reduce the incidence of DVT is unknown. Thus, the hypothesis was that EFM would reduce the incidence of DVT during leg immobilization after ATR surgery.

The primary aim of this randomized controlled trial was to assess the efficacy of EFM to reduce the DVT incidence after ATR surgery, at 2 and 6 weeks postoperatively, compared to treatment-as-usual, i.e., 2 weeks of plaster cast followed by 4 weeks’ orthosis immobilization. The secondary aim was to evaluate the effect of patient extrinsic factors (amount of weightbearing, number of daily steps) as well as patient intrinsic factors (age and BMI) on the risk of sustaining a DVT.

## Materials and methods

Patients between 18–75 years of age with an acute unilateral Achilles midportion tendon rupture were eligible for inclusion if surgery was performed within 1 week after the injury. The exclusion criteria to participate in this study were: current anticoagulation treatment (including acetylsalicylic acid daily dose of 75 mg or higher), known kidney failure, heart failure with pitting edema, thrombophlebitis, thromboembolic event during the previous 3 months, known malignancy, hemophilia, pregnancy, other surgery during the previous month, inability to follow instructions and planned follow-up at another hospital.

**Fig. 1:**
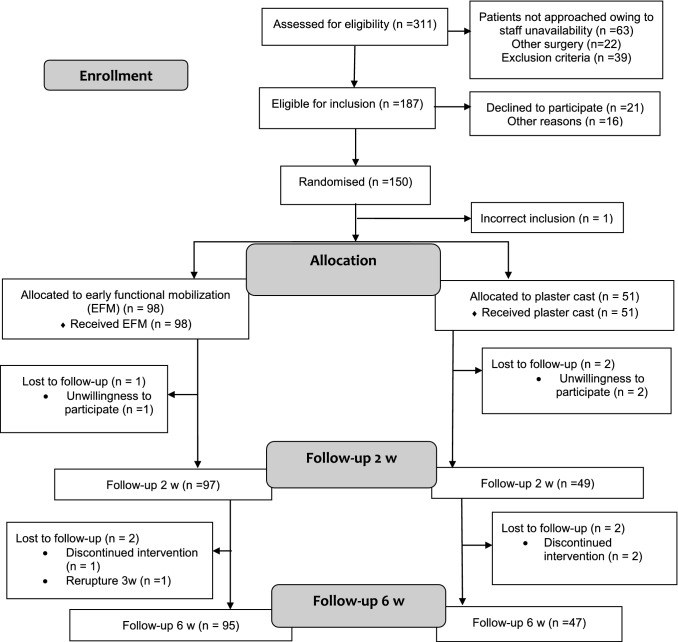
CONSORT flowchart

Between 2013 and 2018, 311 patients with Achilles tendon rupture were screened for eligibility at the Karolinska University Hospital, Danderyds Sjukhus and Södersjukhuset, Stockholm. Of these, 150 patients (114 men and 36 women) were enrolled and randomized postoperatively by a study nurse. Randomization was performed using consecutively numbered sealed envelopes produced by a biostatistician and opened after surgery (Fig. 1). A non-stratified block randomization was used assigning the patient to either direct postoperative EFM or immobilization and non-weightbearing. A computer program was used to generate random numbers in permuted blocks of six and patients were allocated in a ratio 2:1. The Ethical Committee has advised a 2:1 ratio based upon the EFM performing better. Ethical approval was obtained from the Regional Ethical Review Committee in Stockholm, Sweden. (Dnr: 2013/1791-31/3). The study was registered on ClinicalTrials.gov (trial number NCT02318472). All participants received oral and written information about the study procedure and provided written informed consent prior to surgery.

None of the patients were receiving corticosteroid treatments or exhibited active tendinopathy. Characteristics of the study participants are presented in Table 1. There were no significant differences between groups in demographic variables.

**Table 1:** Demographic data on study participants

Variable	EFM (*n* = 98)	Control (*n* = 51)
Age (years), M (SD)	39.2 (8.1)	40.2 (8.3)
Range	(23–64)	(23–58)
Gender (M/F), *n* (%)	75/23 (77/23)	40/11 (78/22)
BMI (kg/m^2^), M (SD)	25.1 (2.8)	25.0 (2.6)
Smoker no/yes/snuff, *n* (%)	75/3/20 (77/3/20)	43/0/7 (86/0/14)
Injured side, R/L, *n* (%)	46/51 (47/53)	24/26 (48/52)
Time to surgery (HH: mm), M (SD)	91:31 (41:25)	89:08 (38:03)
PAS before injury, M (SD)	4.65 (1.0)	4.57 (1.0)

*M* mean, *SD* standard deviation, *EFM* early functional mobilization, *BMI* body mass index, *HH* hours, *mm* minutes, *PAS* Physical Activity Scale

### Surgical procedure

On an outpatient basis, a standardized surgical procedure according to a prespecified protocol was performed, using the modified Kessler suture technique [7]. The surgical procedures were performed by several orthopedic surgeons at one university hospital.

Patients were operated on in prone position without the use of a tourniquet. Local anesthetics were infiltrated in skin, subcutis and peritendinous space with 20 ml of Marcain^®^adrenaline 5 mg/ml + 5 µg/ml (AstraZeneca, London, UK). A longitudinal 5–10-cm skin incision was made over the medial border of the Achilles tendon, and the paratenon was incised in the midline. The tendon stumps were sutured end-to-end using a modified Kessler suture technique with two 1-0 polydioxanone (PDS II) sutures [7]. Thereafter, the paratenon and fascia cruris were sutured separately using 3-0 Vicryl, and the skin was closed with 3-0 Ethilon (Ethicon, Somerville, NJ, USA).

### Postoperative medication

Patients were prescribed acetaminophen 500 mg/codeine 30 mg to cope with the postoperative pain. No pharmacological anti-inflammatory or thromboprophylactic drugs were administered to the patients postoperatively.

### Postoperative regime

The intervention group received early functional mobilization (EFM), which was initiated directly postoperatively in the ward. An orthosis (VACO^®^ped, OPED Gmbh, Germany) with adjustable range of motion in the ankle joint was used. During the first 2 weeks postoperatively, 15°–30° of plantar flexion was allowed. At 2 weeks postoperatively, the range of motion was increased to 5°–30° of plantar flexion for the remaining 4 weeks. Full weightbearing with crutches and plantar flexion exercises was encouraged directly after application of the orthosis.

The control group received treatment-as-usual in a non-weightbearing below-knee plaster cast with the ankle in approximately 30° of plantar flexion applied in the outpatient clinic by an orthopedic cast technician, shortly after the completion of surgery. At 2 weeks postoperatively, the cast was replaced by an orthosis (Aircast^®^ AirSelect™ Elite, DJO, Vista, CA, USA) with three heel wedges for the remaining 4 weeks of immobilization. Every consecutive week, a heel wedge was removed. Full weightbearing with crutches was allowed after application of the orthosis.

The remaining 4 weeks, when both groups were using different orthosis treatments, the patients were instructed to remove the orthosis when seated and to perform several repetitions of active plantar flexion movement (without resistance) from neutral to maximum plantar flexion several times per day. They were allowed to exercise on a stationary bike when wearing the orthosis.

### Follow-up evaluations

#### Assessment of deep venous thrombosis (DVT)

At 2 and 6 weeks postoperatively, all patients were screened for DVT in the injured leg using unilateral compression duplex ultrasound (CDU). A trained nurse or an experienced ultrasonographer, blinded to the treatment regimens, performed all the CDU scans using a Philips CX 50 ultrasound machine (Philips Medical Systems, Andover, MA). The standard procedure included evaluation of all deep proximal and distal veins, including muscle veins, as well as vena saphena magna. The criteria for DVT diagnosis and the diagnostic procedure have been described earlier [14]. Proximal DVT was defined as a thrombosis that involved the popliteal vein or any more proximal veins, with or without involvement of the calf veins. It was not recorded whether a DVT was symptomatic or asymptomatic since it earlier has been demonstrated difficult for clinicians to differentiate between the pain of the rupture or operation and pain that could be caused by a DVT [7, 15].

#### Self-reported diary

At home, from the day after surgery, patients in the EFM group completed a diary on estimated daily weightbearing load, number of steps/day with a pedometer for the first 2 weeks and evaluation of pain on a visual analogue scale (VAS) daily during the first week [2]. Patient self-reported loading has earlier been found to significantly correlate to the plantar force measurements [2]. Patients in the control group completed the pain registration during the first postoperative week.

### Statistical analysis

The power calculation was based upon data from a recent study reporting a 50% rate of CDU-verified DVT after ATR surgery [7]. EFM was estimated to result in a 50% risk reduction [6]. Sixty-three patients in each group were required to detect a difference of 25% in the incidence of DVT (two-sided type-I error rate = 5%; power = 80%). To counteract dropouts, 150 patients were included. On recommendations from the ethical committee, a ratio of 2:1 was chosen, since our hypothesis was that the EFM group would perform better.

The statistical analyses were based on intention-to-treat. Descriptive data were reported as mean, median, range and frequency. Categorical variables, e.g. differences between the EFM- and control group in the occurrence of DVT, were analysed with Pearson’s *χ*^2^ test. The continuous variables were not severely skewed and thus, and difference between groups in these variables were analysed with a parametric Student’s *t* test.

Relationships between variables and outcome were expressed as Pearson’s correlation coefficients. This was done to investigate the unique relationships between the independent variables (gender, age, BMI, nicotine usage and loading) and the dependent variable (DVT). A logistic regression enter analysis was used in the risk factor analysis. Only variables that had a significant correlation with the dependent variable were included in the logistic regression analysis independent of treatment group. Relationships were expressed as odds ratios (OR) with 95% confidence intervals (CI). The level of significance was ≤ 5% in all analyses. All data were analysed in SPSS version 25 (IBM SPSS, Armonk, NY, USA).

## Results

In total 149 patients, with a mean (SD) age of 39.6 (8.1) years were included in an intention-to-treat analysis, of these patients, 142 completed the 6 weeks’ follow-up (Fig. 1).

### Deep venous thrombosis (DVT)

At 2 weeks, 43/145 (30%) of the patients in total demonstrated a CDU-verified DVT, with 28/96 (29%) in the EFM group, and 15/49 (31%) in the control group (n.s). In the control group, one patient sustained a pulmonary embolism 3 days postoperatively and one patient had a proximal DVT, in the popliteal vein at 2 weeks. The remaining DVTs detected at 2 weeks were diagnosed as distal DVTs.

At 6 weeks, 49/143 (34%) of the patients experienced a DVT, with 35/94 (37%) of the patients in the EFM and 14/49 (29%) in the control group (n.s). Ten of the DVTs diagnosed were newly developed and 39 DVTs were remaining from the 2 weeks control. Three patients with CDU-verified DVTs at 2 weeks were at 6 weeks diagnosed without a DVT and one patient lost to follow-up. Of the new DVTs, eight were detected in the EFM group and two were found in the control group. At 6 weeks, one patient in the EFM group sustained a proximal DVT, in the popliteal vein.

The total incidence of DVT at 2 or 6 weeks was 53/144 (37%), of whom 36/95 (38%) were found in the EFM and 17/49 (35%) in the control group (n.s) (Table 2).

**Table 2:** Total incidence of deep venous thrombosis (DVT)

	EFM	Control	*p* valueª
Number of DVTs at 2 weeks	28/96 (29%)	15/49 (31%)	n.s
Number of DVTs at 6 weeks	35/94 (37%)	14/49 (29%)	n.s
Number of DVTs at 2 or 6 weeks	36/95 (38%)	17/49 (35%)	n.s

*EFM* early functional mobilization, *DVT* deep venous thrombosis

ªChi squared test

### Adverse events

One patient sustained a partial rerupture at 3 weeks postoperatively due to slipping when not wearing the orthosis. The patient was successfully re-operated but was excluded from further participation in the study (Fig. 1). Adverse events are reported in Table 3.

**Table 3:** Adverse events

	EFM	Control
Rerupture, *n*	1/98	0/51
Superficial infections, *n*		
2 weeks	1/98	0/51
6 weeks	1/98	1/51
Deep infections, *n*	1/98	0/51

*EFM* early functional mobilization

### Patient-reported loading, pain and steps/day in the EFM group

The patient-reported loading (i.e., in % of full weightbearing being 100%) in the EFM group was low. At the end of week 1 (day 7), 32/92 (35%) of the patients in the EFM group reported loading higher than 50%. The number of steps, loading and pain are reported in Table 4. Patients in the EFM group with less experience of pain during activity the first postoperative week exhibited significantly higher patient-reported load, the correlation coefficients varied between − 0.23 (*p* = 0.028) and − 0.48 (*p* < 0.001).

**Table 4:** Self-reported diary

	EFM (*n* = 98)	Control (*n* = 51)	*p* value
Pain assessment			
Rest, week 1, mean (SD)	20.3 ± 15.8	12.7 ± 14.3	0.023
Activity, week 1, mean (SD)	40.1 ± 21.8	27.2 ± 17.5	0.001
Step counts			
Week 1, median (IQR)	942 (472–2491)	N/A	
Week 2, median (IQR)	2001 (1058–3222)	N/A	
Self-reported loading			
Week 1, median (%)	25	N/A	
Week 2, median (%)	60	N/A	

*EFM* early functional mobilization, *IQR* interquartile range, *N/A* not assessed

### Risk factors of DVT

The relationship between patient characteristics (age, gender, BMI, nicotine usage), pain and DVT was examined. Higher age (*r* = 0.25, *p* = 0.003) and BMI > 26 (*r* = 0.21, *p* = 0.013) were the only factors significantly correlated with higher risk of experiencing a DVT at 2 weeks independent of treatment group. Logistic regression confirmed age and BMI as two independent risk factors of sustaining a DVT, with 2.3 higher odds in patients with a BMI of more than 26 (OR 2.29; 95% CI 1.07–4.91) and 1.07 higher odds per each increased year of age (OR 1.07; 95% CI 1.02–1.12).

**Fig. 2:**
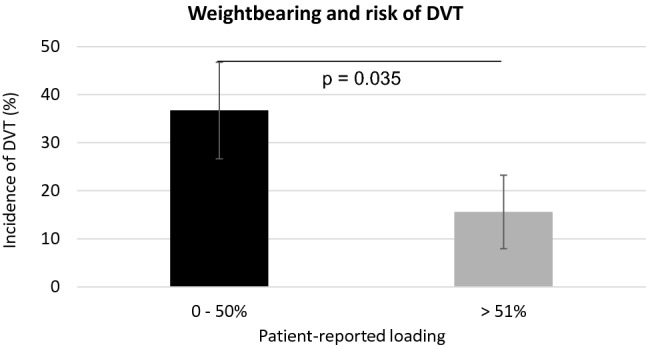
Weightbearing and risk of DVT. *DVT* deep venous thrombosis

The number of steps and weightbearing status were included when analyzing the risk factors for DVT in the EFM group separately. This analysis demonstrated that average daily load of 50% or less at day seven (*r* = 0.22, *p* = 0.035) (Fig. 2) in addition to higher age (*r* = 0.22, *p* = 0.029) and BMI > 26 (*r* = 0.26, *p* = 0.010) were significantly correlated with increased risk of suffering a DVT in the univariate analysis. Logistic regression corroborated that in addition to age and BMI > 26, loading ≤ 50% during the end of week 1 was an independent risk factor associated with 4.3 higher odds of sustaining a DVT (OR = 4.29; 95% CI 1.28–14.3).

## Discussion

The most important finding of the present study was that EFM in an ankle-mobile orthosis versus conventional lower limb plaster cast followed by ankle-stable orthosis immobilization after ATR surgery does not significantly reduce the incidence of DVT. Additionally, three independent risk factors of DVT during limb immobilization were established: age, BMI > 26, and ≤ 50% of weightbearing in the EFM group. According to the novel findings of this study, it can, therefore, be suggested that for prevention of DVT, patients should attain to load half their bodyweight on their lower limb 1 week after surgery.

EFM, i.e. early weightbearing and ankle motion, has increased in popularity and shown greater patient satisfaction compared to conventional leg immobilization in ATR patients [3, 12, 17, 18]. EFM has also been speculated to decrease the risk of DVT, however, without preexisting evidence. The primary aim of this trial was to assess the efficacy of EFM to reduce the DVT incidence after ATR surgery, at 2 and 6 weeks postoperatively. The main findings of no difference in the risk of DVT, neither at 2 nor at 6 weeks, between EFM and conventional leg immobilization suggest that further developed EFM or other means of DVT prevention should be pursued. Furthermore, the high overall risk of DVT, 37%, at 2 and 6 weeks found in this study confirms data from earlier studies [4, 15] and highlights the need of novel DVT preventive interventions in ATR patients [7].

The similar DVT incidence in the two groups at 2 weeks postoperatively, at around 30%, was unexpected given that the EFM group had the possibility to bear weight and move their ankle while the control group was immobilized in a plaster cast. Our finding that the patients in EFM group reported relatively low load in the orthosis, 25–60%, during the first 2 weeks may be one explanatory factor for the ineffectiveness of EFM to reduce the risk of DVT. This conclusion is supported by the demonstration that venous blood flow is reduced during only partial weightbearing [5].

The non-significant difference in DVT incidence at 6 weeks was more expected since both groups were allowed weightbearing in an orthosis. The EFM group was, however, allowed partial ankle motion within the orthosis, which was not the case in the control group. An earlier study has demonstrated that ankle movements during patient lower limb immobilization increases venous return [9]. Our findings, however, suggest that allowing ankle motion within an orthosis is not enough to reduce the risk of DVT, at least when patients are partially weightbearing.

The second main finding of this study pertains to the low amount of weightbearing in the EFM group and that patients who loaded their injured limb less than 50% at the end of week 1, exhibited 4.3 higher odds in the risk of developing a DVT before 2 weeks. The finding is in agreement with data demonstrating increased venous return during more than 50% of weightbearing [5].

Notably, the amount of weightbearing was an independent risk factor for sustaining a DVT. Therefore, the results of this study would seem to suggest that specific recommendations on the amount of weightbearing should be delivered to patients receiving EFM. Whether such advice to leg immobilized patients would be effective for the prevention of DVT warrants further studies. However, it should be mentioned that the patients in this study were informed to weight bear as tolerated and that loading their injured leg directly in the orthosis was safe.

This study also sought to examine whether specific risk factors, which therapists can have an influence on, were related to less amount of weightbearing. The patients’ experience of pain was related to the degree of loading. This observation suggests that adequate pain control to achieve at least 50% of loading at the end of week 1 could be a strategy in the rehabilitation plan to potentially lower the risk of DVT. Our present data could, however, not verify that pain was correlated to the incidence of DVT.

Interestingly, when examining risk factors for DVT, it was found that the number of daily steps taken did not correlate with the incidence of DVT. This observation suggests that the number of daily steps taken does not influence the venous blood flow, but rather the amount of loading. Therefore, postoperative pain medication and patient recommendations should focus on weightbearing instead of long walks.

The finding that patients with BMI over 26 exhibited an almost threefold increased odds for sustaining a DVT at 2 weeks was supported by the literature and may reflect that specific interventions should be initiated for obese patients [20, 22]. Moreover, the observation that the risk of DVT increased by 7% per each increasing year of age, is confirmed by earlier studies [1, 11, 20]. The finding also indicates that older age together with BMI should be considered while deciding DVT preventive interventions during leg immobilization [11, 20, 22].

This is the first prospective randomized study to study DVT rate during EFM. The non-effectiveness of EFM can partly be explained by low weightbearing during early orthosis mobilization. Thus, one potential limitation of our study might be that the patients were not encouraged enough to bear weight in the orthosis. The patients were, however, instructed that weightbearing was not harmful and additionally were allowed 1 h of daily unloaded plantar flexion exercises without the orthosis. CDU scans do not give 100% correct diagnoses. The finding that three patients with CDU-verified DVTs at 2 weeks at 6 weeks were diagnosed as not having a DVT may be explained by either that the CDU-verified DVTs have been misdiagnosed or that the DVTs have been resolved. The strengths of our study are the relatively large sample-size and a meticulous assessment of DVT as well as analyses of risk factors for the development of DVT during leg immobilization.

The current study indicates that patients should aim to load 50% of their body weight or more 1 week after surgery to minimize the risk for DVT.

## Conclusions

Patients with lower limb immobilization after surgical repair of ATR do not have reduced rates of DVT when using EFM, including early weightbearing and ankle range of motion, compared to lower limb plaster cast followed by orthosis immobilization. Postoperative orthosis leg immobilization is still linked with a high incidence of DVT, which is mainly explained by the three independent risk factors: decreased weightbearing less than 50%, increased age and BMI > 26.
